# Progressive Trends in Hybrid Material-Based Chemiresistive Sensors for Nitroaromatic Compounds

**DOI:** 10.3390/polym14214643

**Published:** 2022-10-31

**Authors:** Gaurav Awasthi, Ritika Sharma, Subramanian Sundarrajan, Seeram Ramakrishna, Pawan Kumar

**Affiliations:** 1 Materials Research Application Lab (MARL), Department of Nano Sciences & Materials, Central University of Jammu, Jammu 181143, India; 2 NUS Centre for Nanotechnology and Sustainability, Department of Mechanical Engineering, National University of Singapore, Singapore 11758, Singapore; 3 Department of Prosthodontics, Saveetha Dental College and Hospitals, Saveetha Institute of Medical & Technical Sciences, Saveetha University, Chennai 600077, India

**Keywords:** hybrid materials (HMs), inorganic-organic materials, coordination polymers, carbon materials, chemiresistor

## Abstract

In the last decades, development of hybrid materials, especially inorganic–organic materials, coordination polymers, conducting polymers, carbon materials, and many more, has produced breakthroughs in diverse applications. Various advance materials have been reported in the literature using metal organic frameworks (MOFs), which compensate for the limitations of sensors. Diverse combinations of HMs not only offer excellent features, but also give a ray of hope for unprecedented advances in materials in different research areas, such as sensing, energy storage, catalysis, non-linear optics, drug-delivery systems, gas storage, etc. Chemiresistor sensors are a core enabling sensor technology and have led to much progress in the field of material science. Here, we have reviewed the recent progress in chemiresistive sensors based on HMs for nitroaromatic compounds, which could be beneficial for researchers that explore this field further. We have put emphasis on sensing mechanisms and the performance of diverse HMs for nitroaromatic sensing applications including pesticides, pollutants, explosives, polycyclic aromatic hydrocarbons (PAHs) and persistent organic pollutants (POPs). In the end, we explored opportunities, challenges, and future perspectives in this emerging field.

## 1. Introduction

### 1.1. Background of the Hybrid Materials

Hybrid materials, in accordance with the International Union of Pure and Applied Chemistry, are materials made up of a combination of inorganic and organic components, or a combination of both with sizes varying from a few nanometers to tens of nanometers, and they are developing into a very powerful and promising category of materials [1,2]. Although the development of organic-inorganic hybrid materials attracted both scientific and industrial attention in the early 1940s, it is worth noting that the study of hybrid materials underwent its first major evolution mostly between the 17th century and the modern era [3,4]. The earliest period, which proved the existence of hybrid materials, extended from prehistory (20,000 years ago) to the 10th century AD. It can be demonstrated with examples such as the bleaching agents that were based on clay and used in ancient Rome or Cyprus. The hybrid clays were utilized to shape and encase Chinese porcelain known as “egg shell”, and the Maya Blue or Prussian Blue pigments. In the case of natural materials, mostly, the organic component holds the inorganic constituents and/or soft tissue together, while the inorganic part gives them mechanical strength and a structure as a whole [5]. In actuality, these natural hybrid materials are frequently the most integrated intelligent systems, which are skilled in striking trade-offs between various tasks such as mechanical behavior, density, controlled permeability, color, and hydrophobicity [3]. In addition, man-made hybrid materials have existed since the beginning of time; for instance, an intercalated organic color compound called Maya blue found in clay minerals, demonstrates the utilization of hybrid materials in the old days [2]. Hybrid materials are divided into four categories [6]. They are

(i) Composites: matrix and micron-level dispersion constituting the material mixture.
(ii) Nanocomposites: combination of comparable types of materials at the sub-micron scale.
(iii) Hybrids: a sub-micron-scale combination of several materials.
(iv) Nanohybrids: composite, nanocomposite, hybrid, and non-hybrid materials that have been combined at the atomic or molecular level via chemical bonding.

**Figure 1:**
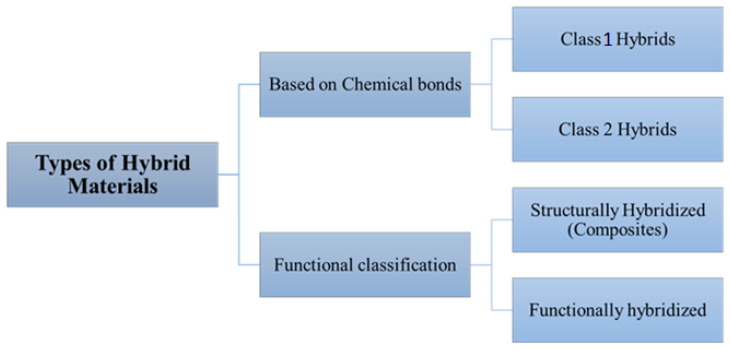
Types of hybrid materials.

Based on chemical bond strength, HMs are classified into Class 1 hybrids (hybrids with weak bonding such as van der Waals forces, weak electrostatic interactions, and hydrogen bonding) and Class 2 hybrids (strong interactions/Covalent bonding between components) (Figure 1). The organic–inorganic hybrids can be categorized based on their interfaces as Class 1 materials, which interact weakly and are linked by blends or interpenetrating networks. On the other hand, Class 2 materials interact through strong chemical bonds, and they are linked by covalently connected polymers. An example of a Class 1 hybrid material is a binary blend of poly (2,6-dimethyl-1,4-phenylene ether) (PPE)/poly (styrene-co-acrylonitrile) (SAN), and an example of a Class 2 hybrid material is obtained by the interaction of three organic components, PPE, SAN and SBM (polystyrene-block-polybutadiene-block-poly (methyl methacrylate)). HMs that are structural composites can be further classified as either single-layer (continuous or discontinuous fibers) or multilayer (laminates) [7]. Metal organic frameworks (MOFs) have been combined with other functional materials such as metal nanoparticles (NPs), polymers, and other MOFs to create MOF-based hybrid materials. Hybrid materials based on covalent organic frameworks (COF) have also been designed [8]. The introduction of MOFs with high electrical conductivity or intrinsic charge mobility presents the possibility for creation of new types of MOF-based sensing devices. In the new era, two-dimensional (2D) and three-dimensional (3D) types of MOFs are utilized in chemiresistive sensors. The highest conductivity values were found in 2D MOFs. It is due to the prolonged conjugation and in-plane charge delocalization in the 2D sheets, which were mediated through electronic communication via the metal nodes [9,10]. For the construction of reliable chemiresistive sensors, it is also important to have a hierarchical pore structure, higher thermal and chemical stability, and strong bonds between each of the analytes. All of these properties are found in MOFs as well as in COFs [11,12]. Considering their functional groups, holes, and highly organized porosity structure, COFs offer a huge active site in which to insert electroactive molecules. Additionally, the stability of electrochemical sensors is increased by their improved biocompatibility [12].

### 1.2. History of Chemiresistive Sensors

**Figure 2:**
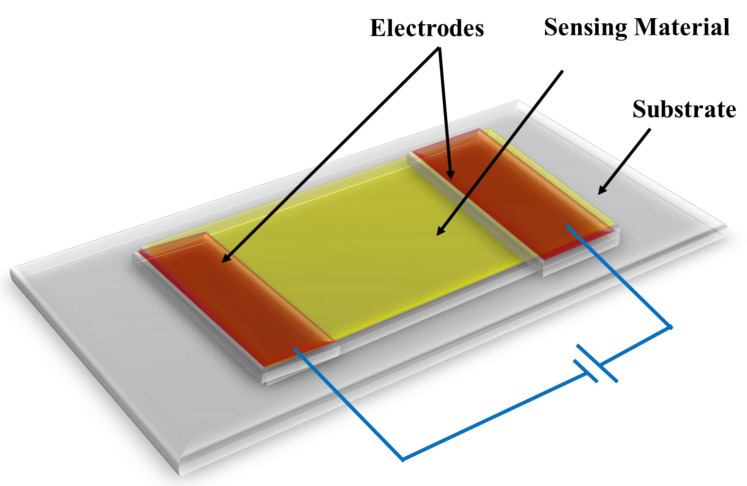
Schematic representation of chemiresistor sensor.

Chemiresistive sensors are conductive materials with a built-in resistance or conductance that changes in response to analyte binding. The resistance change occurs due to either electron or hole transfer induced by the surface reaction between the analyte and the sensing substance. The analyte interacts with the sensing material via covalent bonding, hydrogen bonding or molecular recognition. Wohltjen and co-workers were the first to coin the term “chemiresistor” in 1985 [13]. They investigated copper phthalocyanine complex as a chemiresistive material. At room temperature, it was found that the resistance of the complex decreased when ammonia vapor was present. In 1970, a carbon monoxide detector using powdered SnO_2_ became the first commercialized metal oxide chemiresistive sensor [14]. Since the mid 1990s, mixed-metal oxide chemiresistor sensors have been commercially available for medical, industrial, and air quality monitoring applications [15]. Metal oxide-based chemiresistive sensors are generally gas sensors that can detect oxidizing as well as reducing gases. Metal oxide chemiresistive sensors require high operating temperature, i.e., 200 °C or higher in order to remove an activation energy barrier for resistivity to change [16]. After metal oxides, conductive polymers are the second most researched material as chemiresistive sensors. The most cited chemiresistive materials include metal oxides (MO_x_), metallic nanoparticles, conductive polymers, and carbon-based nanomaterials such as carbon nanotubes and graphene [17]. Composite chemiresistive sensors have recently been developed by integrating two high-performance materials. These composite materials exhibit a significant boost in sensing properties when compared to pristine materials. A schematic diagram of a chemiresistor sensor is displayed in Figure 2 [16].

### 1.3. Broadening of Nitroaromatic Compounds

Nitroaromatic compounds (NACs) are one of the most prevalent and significant classes of industrial chemicals currently in use. The chemical structure of NACs includes one or more nitro groups, and they are aromatic in nature. These compounds are synthetic as well as naturally occurring compounds. Nitroaromatic compounds can occur in both aqueous and atmospheric conditions. Beginning in the early 19th century, the chemistry of nitro compounds was developed, and in the 20th century, it was combined with organic chemistry [18]. Nitro compounds are crucial as synthetic intermediates and building blocks for the synthesis of frameworks for medicines, agrochemicals, dyes, and explosives [18]. Nitro groups have high electronegativity that causes delocalization of the π-electron to accomplish its own charge deficiency [19]. The primary reaction step is nitration, which is generally used to synthesize nitroaromatic compounds. When sulfuric and nitric acids are mixed, nitronium ions (NO^2+^) are produced. These ions are then used to react with aromatic substrates in an electrophilic substitution [19]. Several nitroaromatic compounds have been used in high-energy explosives due to the nitro group’s peculiar chemistry. The nitrogen atom rapidly accepts electrons in this oxidation state (III), allowing explosives based on nitroarene to serve as self-oxidants. As a result, due to detonation of an explosive charge, energy is quickly released from the molecules. HNO_3_ alone or in combination with H_2_SO_4_ is used in traditional nitration procedures, and this method has remained unchallenged for more than 150 years [18].

The most commonly used NACs include 2,4,6-Trinitrobenzene (TNB), 2,4,6-Trinitrophenol (TNP), 2,4,6-Trinitrotoluene (TNT), Dinitrobenzene (DNB), Dinitrotoluene (DNT), Dinitronaphthalene (DNN), 1,3,5-Trinitroso-1,3,5-triazinane (RDX), Dinitrophenol (DNP), Nitrobenzene (NB), Nitroaniline (NA), and Nitrocatechol (NC). NACs can be used as starting materials in the chemical synthesis of a wide range of substances, including explosives, pesticides, dyes, drugs, cosmetics, preservatives, paints, corrosion inhibitors, gasoline additives, and other industrial chemicals. The nitro group has some special properties that make it useful in this regard [20].

Picric acid or 1,3,5-trinitrophenol (TNP) was initially developed as a yellow fabric dye in 1771, and it is now used in explosive shells [21]. Toluene and nitric acid are combined to create dinitrotoluene (DNT). Although DNT exists with six isomers, most of the information is relevant to 2,4-DNT and 2,6-DNT [22]. Mononitrotoluene, DNT, and TNT are the products sequentially produced by the nitration of toluene. 2,4-DNT is synthesized through the nitration of 4-nitrotoluene [23]. Explosive DNT is used to make smokeless powders, as a rocket propellant plasticizer, and as a gelatinizing and waterproofing agent [24].

**Figure 3:**
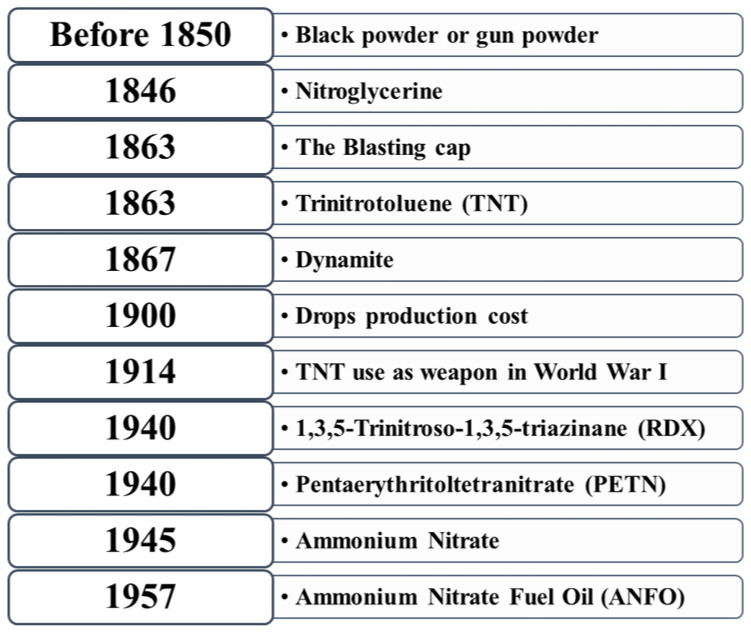
Chronological history of explosives [25].

A chronology of nitro compounds as explosive is presented in Figure 3. In 1867, dynamite was invented. TNT was used as a weapon in World War I in 1914 as it had more advantages over dynamite since the shock waves produced by TNT could rupture the steel on armor-plated vehicles. During World War II, two new explosives were introduced, RDX and PETN (penta erythrito tetranitrate). RDX was renamed as Composition Four or C-4 explosive. In 1945, ammonium nitrate, as an inexpensive fertilizer, was manufactured and shipped to Europe for enriching depleted farm soil. In 1957, ammonium nitrate fuel oil was developed as an explosive [25].

## 2. Prerequisites for Chemiresistive Sensors

A simple chemiresistor is made up of a sensing material that coats a group of interdigitated electrodes or fills the space between two electrodes. It measures the resistance lying between the electrodes. As analytes interact with the sensing material, the intrinsic resistance of the sensing material can be altered in their presence. These interactions result in changes in measured electric properties such as resistance, which can be used to determine if an analyte is present or not, as well as its quantity if present.

**Figure 4:**
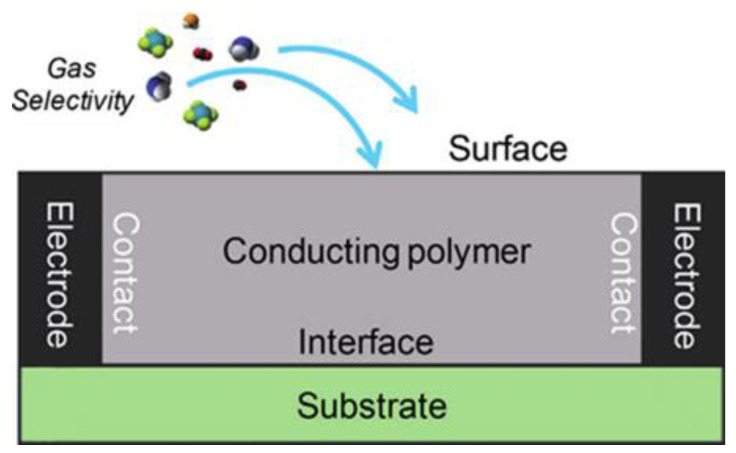
The chemiresistor sensor idea is illustrated schematically for the purpose of selectively detecting chemical sensing materials using chemically sensitive CPs. Reprinted from [27].

It is advantageous to generate π-stacking complexes with electron-rich fluorophores due to NACs’ electron-deficient characteristic. It can be used to detect them using chromo-fluorogenic probes. Chemical sensors offer unique techniques for the fast detection of ultratrace NACs in explosives and can be integrated with small microelectronic systems [26]. Single-walled carbon nanotubes (SWCNTs) are a desirable type of chemiresistor due to their low-cost synthesis, ability to operate at ambient temperature, and extremely low power needs. The principle of a chemiresistor formed on chemically sensitive conducting polymers for the specific detection of chemical sensing substances is schematically illustrated in Figure 4 [27].

**Figure 5:**
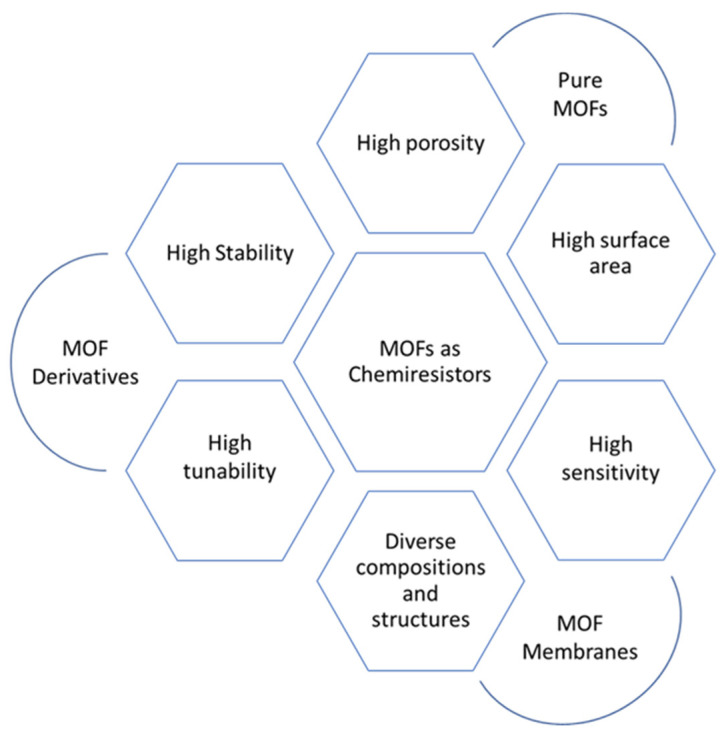
MOFs as chemiresistor sensors, with their properties and different forms.

More recently, research on chemiresistor sensors based on MOFs has been gaining more attention due to their excellent properties, including high porosity, high surface area, chemical and thermal stability, and stable luminescent/electrochemical nature [28]. All of these properties have been summarized in Figure 5. Importantly, MOFs constructed from periodic table Group IV elements, i.e., metals such as cerium, zirconium, and hafnium, have become of particular interest. It is due to their notable chemical stability in water and their operation in aqueous media. Many research reports have been published on similar MOFs for chemosensors since 2013 [29]. Additionally, adding MOFs to a composite of active sensing materials will enhance the performance of chemiresistive sensors. Numerous research initiatives have been implemented in this regard to develop different electrically conductive MOFs as well as materials generated from MOFs for their usage in a variety of applications, including chemiresistive sensors [28]. For instance, chemically resistive sensors for the detection of CO_2_, NO_2_, and SO_2_ gases can be produced with UiO-66 and its derivatives. Due to their low electrical conductivities, the resistive responses of such Zr-MOFs are still within the range of 10^−10^ Ohm [30].

## 3. Progress on Chemiresistive Sensors for Nitroaromatic Compounds

Many of the chemiresistors based on hybrid materials such as quantum dots, carbon nanotubes, nanosheets, and fibers for sensing of nitroaromatics have been summarized in Table 1. From Table 1, we can conclude that the chemiresistive sensors most used for nitroaromatics are nanosheets and transition-metal doped nanoparticles. TiO_2_ nanosheets and Ni-ZnO were reported for sensing of various explosives such as PNT, RDX and TNT at analyte concentrations as low as 9 ppb.

NACs are a common form of organic pollutant found in the environment [39]. NACs are extensively employed in dyes, fireworks, leather, pharmaceuticals, cosmetics, and agrochemicals (pesticides, herbicides, fungicides). Additionally, they are used as preservatives, paints, antioxidants, gasoline additives, explosives and corrosion inhibitors [20,40]. NACs were also discovered in various food items, e.g., vegetables, grilled and smoked meats, tea, coffee, spices, fresh and cured meat items, oils, and beverages [39]. As per the available literature, approximately 65,000 nitroaromatic pollutants are released from various chemical industries/sources [41] into the environment. In the sensing of nitroaromatic compounds, electrophoresis (CE), spectrometric methods such as Raman spectroscopy (RS) and ion mobility spectrometry (IMS), and chromatography techniques such as liquid chromatography-tandem mass spectrometry (LC-MS) and gas chromatography coupled with mass spectrometry (GC-MS) are used. These detection techniques are conventional, complicated, and need a trained person. In recent years, various sensor-based techniques have also been used in the detection of NACs. Amongst them, optical fluorescence sensing is the most common method for the detection of NACs. The fluorescence method is based on the fact that reactions between sensors and analytes change the luminescent spectra [42]. Apart from fluorescence techniques, various other techniques such as colorimetric [43], MIP based [44] sensing of nitroaromatics are also used.

**Figure 6:**
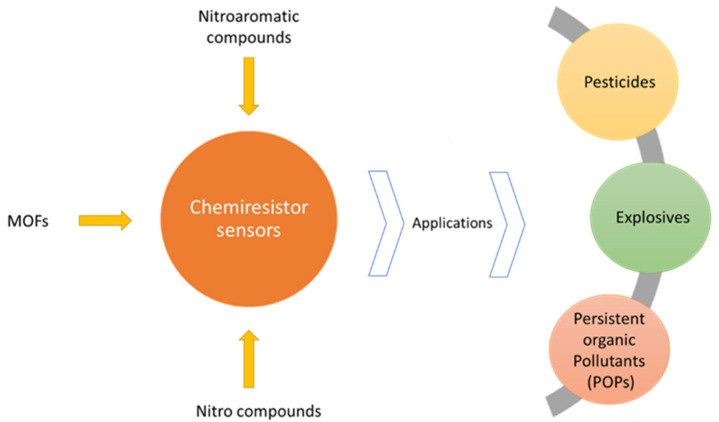
Chemiresistor sensor and its applications.

The chemiresistive sensing technique is an electrical sensing method that is based on resistance (or conductance) change. In the chemiresistive sensor, the flow of holes or electrons generated either by adsorptions or surface reactions of analyte molecules on detecting materials occurs [28]. Chemiresistive sensors provide a number of advantages, including inexpensive manufacturing costs, ease of integration with a variety of electronic devices, and downsizing [28]. Chemiresistive sensors are made up of a thin layer of chemically sensitive material, coated on a conductive electrode platform, which changes its electrical resistance when exposed to a particular analyte. Metal oxide or conducting polymer sheets are commonly used in traditional chemiresistors [45]. Advancement in technologies gave rise to various materials for chemiresistive sensing, such as quantum dots [31], carbon nanotubes [46], graphenes and their oxides [47], hybrid nanoparticles [48], and MOFs [28]. There are different types of nitroaromatic compounds, and they have different chemiresistive sensitivities. They are described in the upcoming paragraphs. Figure 6 shows a chemiresistor sensor and its utility for sensing different materials.

### 3.1. Pesticide Sensors

**Figure 7:**
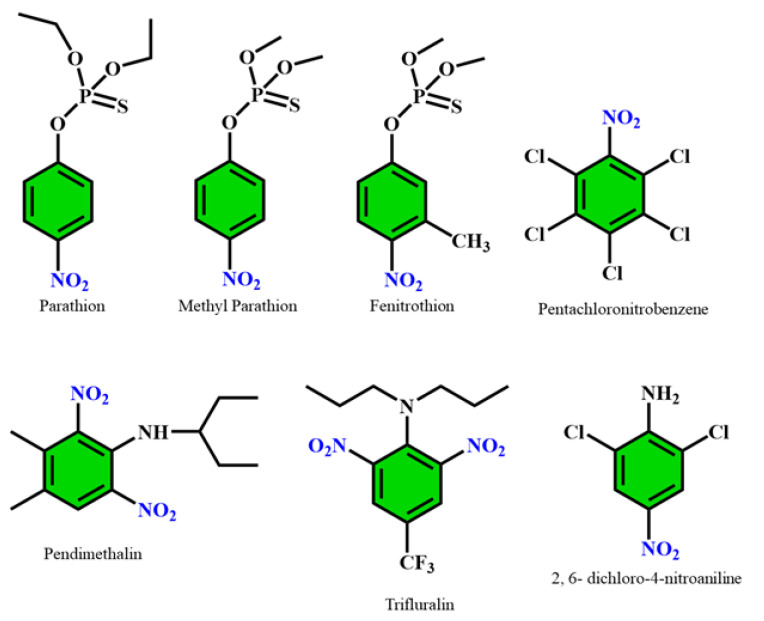
Chemical structures of some nitro group-containing pesticides.

**Figure 8:**
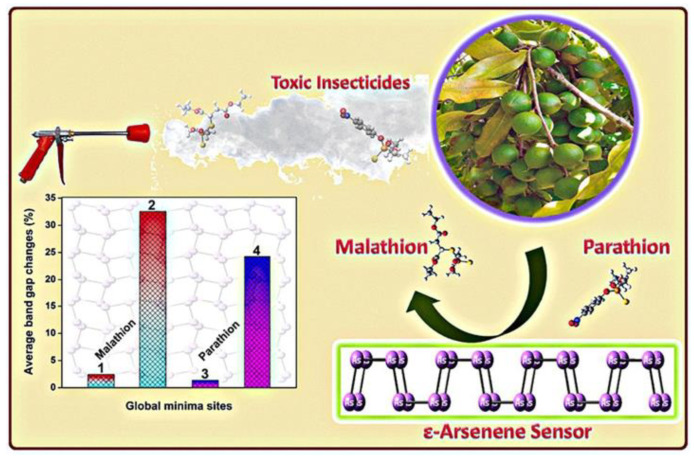
Detection of ethyl parathion—chemiresistive approach. Reprinted with permission from [51].

Pesticides are chemicals that are applied to destroy as well as control pests and weeds. They are commonly employed in agricultural fields to save crops from a range of infections and pests. Excessive uses of pesticides cause several harmful impacts on the environment as well as on living beings [49]. Organophosphate pesticides (OPs) are a varied group of chemicals that include insecticides, fungicides, and herbicides. Several pesticides such as parathion, fenitrothion, methyl parathion, pendimethalin, trifluralin, pentachloronitrobenzene, 2,6-dichloro-4-nitroaniline etc. contain nitro groups in their structures, as shown in Figure 7 [50]. Bhuvaneswari et al. in 2020 presented a density functional theory (DFT)-based chemiresistive approach for the sensing of ethyl parathion [44]. They used a 2D nanomaterial, namely ε-Arsenene nanosheet, for the absorption of target molecules that are preeminent allotropes of arsenene (Figure 8). Several factors, such as the projected density of states (PDOS) spectrum, adsorption energy, electron density, energy band gap, average energy gap, and Bader charge transfer modification, indicate the utility of ε-Arsenene nanosheet as a chemiresistive sensor.

Using dielectrophoretically aligned SWNTs across electrode pairs for real-time detection of paraoxon was shown by Liu et al. (2007). When paraoxon is injected into the sensors, the electrical conductance changes in real time because of enzymatic hydrolysis. Aligned carbon nanotubes are used, as they provide consistent coverage and increase the interfacial contact between the organophosphorus hydrolase (OPH) enzyme and the SWNTs, which can increase sensitivity. However, the non-specific binding (NSB) nature of the enzyme hinders the biosensor from being useful since it may cause enzyme leaching during operating circumstances [52].

### 3.2. Explosive Sensor

**Figure 9:**
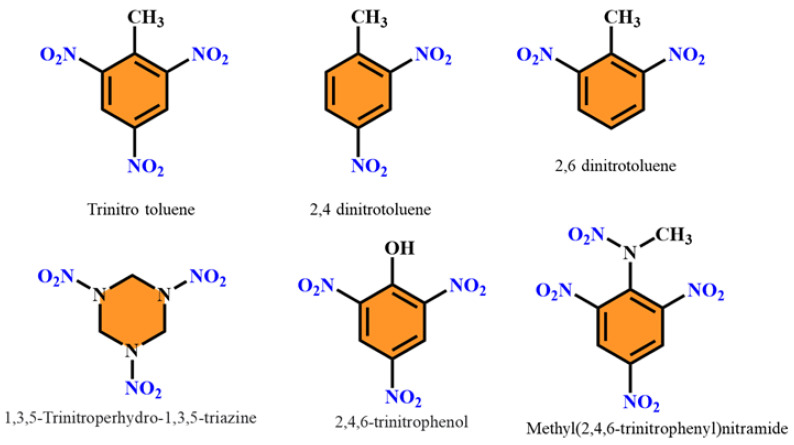
Chemical structures of nitroaromatic explosive compounds.

Due to the escalating terrorism situation, fast and accurate detection of explosives has come to be a major global challenge [33]. In their illicit explosive devices, terrorists employ both less potent homemade explosives made of readily available commercial chemicals as well as powerful military explosives. Military explosives include 2,4,6-trinitrotoluene (TNT), hexogen (RDX), dinitrotoluene (DNT), and other nitro-explosives, and their chemical structures are shown in Figure 9 [33].

To monitor these substances, a variety of analytical methods are available, including Raman spectroscopy, gas chromatography coupled with mass spectrometry (GCMS), ion mobility spectrometry, fluorescence, etc. [53]. Apart from these, trained animals are also used for the sensing of explosive materials [54]. To enable the rapid and precise detection of dangerous compounds in a range of government agencies and public facilities such as airports, railway stations, and bus stations, highly sophisticated systems with high sensitivity, mobility, minimal power utilization, and low cost are needed. Since explosives have low vapor pressure, they are difficult to detect. Vapor detection methods must be capable of detecting extremely low concentrations and/or sampling enormous volumes [55]. Several materials, such as metal organic frameworks (MOFs), quantum dots (QD), graphene, etc., are used to detect nitro explosive in the vapor phase. For the fabrication of chemiresistors, colloidal QD offers various advantages: (a) QD treatment from the solution phase and deposition on various substrates are made possible by the colloidal stability of QD dispersions; (b) the QD’s high surface-to-volume ratio enhances the potential for analyte detection through QD surface chemical modification; and (c) a variety of metal electrodes can be employed since the QD energy levels can be adjusted in accordance with the size-dependent electronic structure [31].

**Figure 10:**
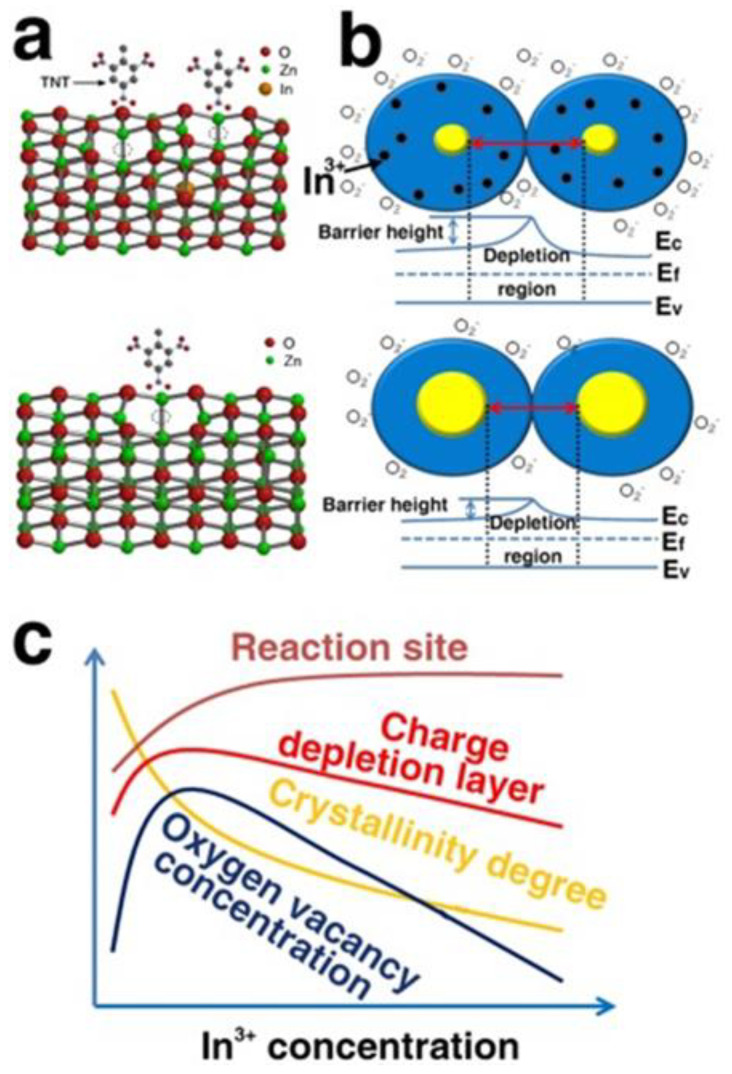
(**a**) Schematic representations of the 5% In-doped ZnO (upper part) and pure ZnO NPs crystal structures (lower part). (**b**) Schematic illustration of sensing on the surface of 5% In-doped ZnO (upper part) and pure ZnO NPs (lower part), and (**c**) possible effects of In^3+^ concentration on the reaction site, the oxygen vacancy concentration, crystallinity degree and charge depletion layer depth. Reprinted with permission from [59].

The EDA-capped PbS QD sensor can efficiently detect nitro-benzene vapor at ambient temperature, with a response of 0.34% measured at an NB concentration of 65 ppb with 2 ppb detection [31]. Graphene-based devices for chemiresistive sensing are particularly intriguing because they have the potential to integrate flexibility with excellent mechanical properties, thermal stability, electrical conductivity, and specific surface area [56]. As the forerunners of the RGO gas sensor, reduced graphene oxide (RGO) sensors could detect warfare chemicals and explosives at parts-per-billion (ppb) levels [57]. Trinitrotoluene (TNT) sensing has been achieved in seawater using graphene nanoribbons (GNRs) and graphene films. Graphene sheets used for chemiresistive detection of NO_2_ showed qualitatively similar conductance variations when exposed to the pseudo explosive 2,4-dinitrotoluene (DNT) [58]. As a result of interaction between its aromatic hexagon structure and the graphene surface, the TNT molecule induces a higher shift in conductance than the nitramines [58]. Ge et al. in 2017 reported a gas sensor with In-doped ZnO nanoparticles, which responded more favorably to saturated nitro-explosive vapors at ambient temperature. In contrast to the pure ZnO nanoparticle-based sensors, responses to DNT, TNT, PNT, RDX and PA were enhanced from 8.5, 22.2, 2.9, 9.8, and 4.9% to the values of 52.9, 54.7, 57.2, 47.4, and 58.3%, respectively. Furthermore, a significantly faster response time (<6.3 s vs. 20–40 s) [59] was observed. The crystal structures of 5% In-doped ZnO and pure ZnO NPs are schematically shown in Figure 10.

**Figure 11:**
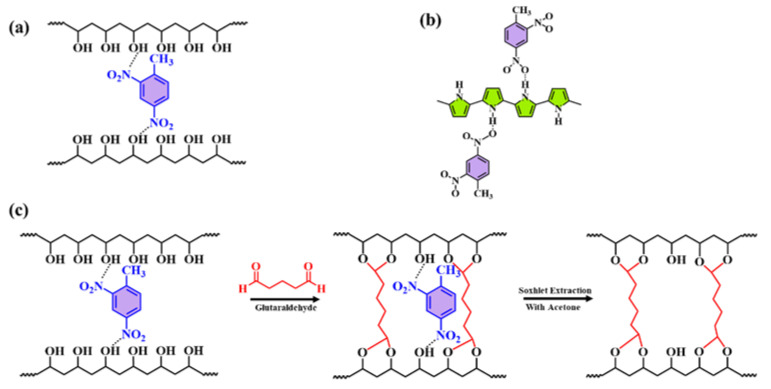
A diagram depicting the interactions of 2,4-DNT with the components of nanocomposite materials (**a**) PVA, (**b**) PPy and (**c**) MIP. Reprinted with permission from [60].

A nanocomposite of polyvinyl alcohol, polypyrrole, and molecularly imprinted polymer (PVA/PPy/MIP) was synthesized and fabricated in order to identify the 2,4-DNT vapor as a non-aromatizing explosive substance. This sensor showed a linear range of response between 0.1 and 70 parts per million [60].The sensor’s ability to detect DNT is due to strong hydrogen bonds that exist between the explosive nitroaromatic material and polypyrrole’s recoverable adsorption, as shown in Figure 11 [60]. Another graphene-based MIP sensor developed by researchers had cavities consistent with nitrobenzene molecules. Methacrylic acid and vinyl benzene were used as the monomers during its synthesis. It was then mixed with graphene to develop a chemiresistor gas sensor from nanocomposite. This sensor responded linearly to concentrations between 0.50 and 60.0 ppm, with a 0.2 ppm sensing limit [61].

Carbon nanotubes (CNTs) were discovered by Iijima in 1991, and they are considered as a perfect sensing component for chemical sensors due to their distinct qualities. CNTs are classified as single-wall carbon nanotubes (SWCNTs), double-wall carbon nanotubes (DWCNTs), and multi-wall carbon nanotubes (MWCNTs) [62]. The SWCNTs fabricated with 1-pyrenemethylamine (PMA), known as PMA-SWCNTs, were used to create a chemiresistive sensor with exceptional sensitivity for TNT detection. Due to the selective interaction between the amino substituent of PMA and TNT, negatively charged complexes were formed, which acted as molecular gates. PMA-SWCNT had an LOD of 10 ppt and a response time of less than 1 min for TNT sensing [37,62]. Another group of researchers fabricated an SWCNT chemiresistive sensor in order to detect nitroaromatic explosives. They fabricated sensors using a porous thin SWCNT film, and it was coated with an oligomer of carbazolylethynylene (Tg-Car) for nitroaromatic explosive sensing [38].

Apart from the above nanomaterials, a dye-doped chemiresistive sensor for TNT sensing was also developed. In 2017, Ghoorchian et al. developed a chemiresistive gas sensor for very sensitive ambient 2,4,6-trinitrotoluene sensing in air. It is based on a modified conducting polypyrrole layer doped with sulfonated dye. They fabricated a sensor via electrosynthesis of polypyrrole (PPy) on Au interdigital electrodes (Au-IDEs) with the exposure of the sulfonated dyes. The films were treated using n-butylamine (nBA). When TNT was absorbed on the film, it formed the Meisenheimer complex. The detection limit for determination of TNT with this sensor was reported to be 0.2 ppb [34].

Zhang and their co-workers in 2019 developed a filter paper-based chemiresistive sensor for nitroaromatic detection. They sprayed polyaniline (PANI) on filter paper and achieved non-contact, rapid sensing of nitroaromatic explosives. They achieved limits of detection of 0.094 ppb for 2,4,6-trinitrotoluene (TNT) and 0.029 ppb for picric acid (PA) [35].

### 3.3. Persistent Organic Pollutant (POP) Sensors

Persistent organic pollutants (POPs) are a category of hazardous synthetic chemicals with a high level of chemical resistance and a long half-life before degrading. They resist chemical, biological, and photochemical degradation and thus pose a huge risk to the environment [63]. Thus, through the Stockholm Convention on Persistent Organic Pollutants, an international agreement was introduced in 2001 to regulate, eliminate, and manage POPs (United Nations Environment Programme, 2001) [64]. Since most of the POPs exist in isomeric forms, development of highly selective systems is very important. A few of examples of POPs include Aldrin, Dieldrin, DDT, Hexachlorobenzene, Chlordane, etc.

A variety of imine-linked covalent organic frameworks (COFs) including triphenylbenzene as an intrinsically luminous platform and a luminescent azine-linked COF that functioned as a docking site to lock guest molecules were used to report the chemo-sensing capabilities for polynitroaromatic compounds. Pablo et al. (2019) reported materials for the chemical detection of contaminants directly in water using pyrene-IMDEA-COF. They reported the disintegration of materials and the formation of stable aqueous suspensions in order to find a number of possible water pollutants, such as nitro explosive compounds and organic dyes. To explore the potential use of COF colloids as chemical sensors, their fluorescence properties were assessed. For a range of organic dyes, the colloidal IMDEA-COF-1 nanolayers exhibited impressive chemical sensing abilities to dyes such as nitrobenzene, dinitrobenzene, methylene blue, janus green, malachite green, bromophenol blue, thionin and crystal violet. They proposed that the mechanism of IMDEA-water COF-1’s colloid pollutant detection was associated with the quenching effect of the interaction between the surface of the colloid’s COF nanolayers and the aromatic portion of the nitro derivative or organic dye [65].

**Figure 12:**
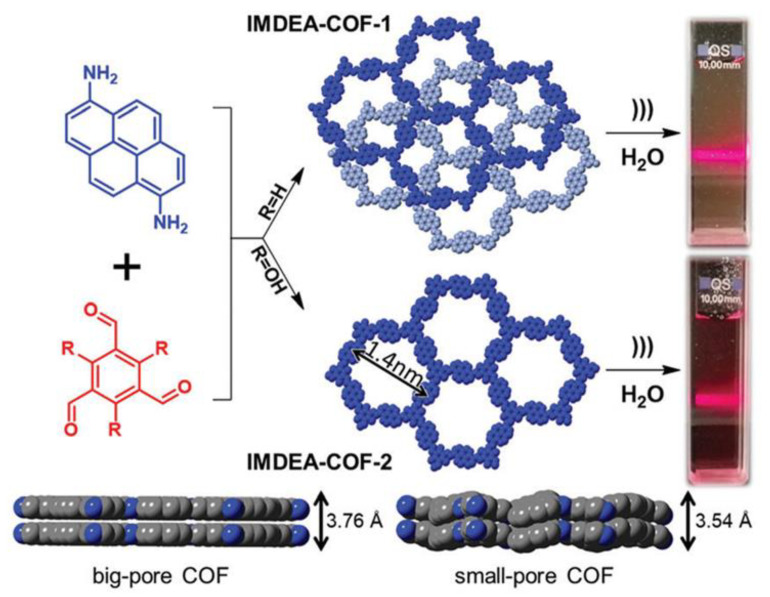
Diagram illustrating the two-step process used to create the water colloids for IMDEA-COF-1 and IMDEA-COF-2. Tyndall effect of the obtained colloids also shown. Reprinted with permission from [65].

Novel MOF-5 covered SERS-active gold gratings were reported by Guselnikova and their co-workers [66]. A schematic of the two-step process used to create the water colloids (Tyndall effect) for IMDEA-COF-1 and IMDEA-COF-2 is shown in Figure 12**.** Growing MOF materials on specifically designed gold gratings has the main benefit of reducing the inhomogeneity caused by the aggregation of metal nanoparticles, which are frequently employed as plasmonic surfaces. The repeatability of SERS signals was subsequently improved. The created platform had a detection limit of 10^−12^ M for two polar organophosphorus insecticides, paraoxon and fenitrothion.

### 3.4. Polyaromatic Hydrocarbon Sensor

**Figure 13:**
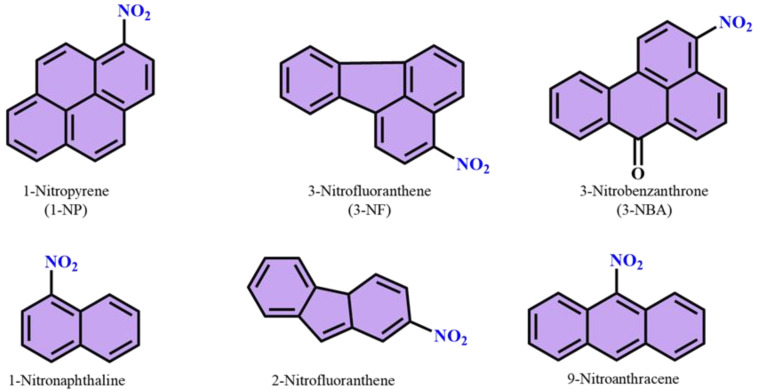
Chemical structures of nitro group containing polyaromatic hydrocarbons (nitro PAHs).

Polycyclic aromatic hydrocarbons (PAHs) that include at least one nitro-functional group on their aromatic benzene ring are called nitro-polycyclic aromatic hydrocarbons (N-PAHs) [67]. The N-PAHs are the main subgroup of PAHs existing in ambient air particles occurring from diesel emissions [68]. One of the most common N-PAHs discovered in diesel emission particles is 1-nitropyrene (1-NP) (DEPs), and it is a main contributor to DEPs’ mutagenicity [68]. N-PAHs can occur through thermal decomposition of organic compounds and are found in toners of photocopying machines, combustion emissions from gas fuel, kerosene heaters, and liquified petroleum, coal fly ash, air particulates, and food. They are largely found in particulate matter in the environment [69,70]. N-PAHs are more highly carcinogenic than PAHs. Even if they are present in smaller trace amounts than their parent substances, N-PAHs can be primarily produced from the same source of PAHs, although secondary production is frequently caused by interactions with OH and NO_3_ radicals [71]. Numerous N-PAHs have undergone significant research into their toxicological properties, including their mutagenicity, carcinogenicity, and metabolism [70]. A study of N-PAHs’ variations in urban Shanghai showed that, under meteorological conditions, the amounts of PAH and NPAH present were both dominated by ambient temperature [72]. Chemical structures of some N-PAHs are shown in Figure 13.

### 3.5. Miscellaneous Sensing

**Figure 14:**
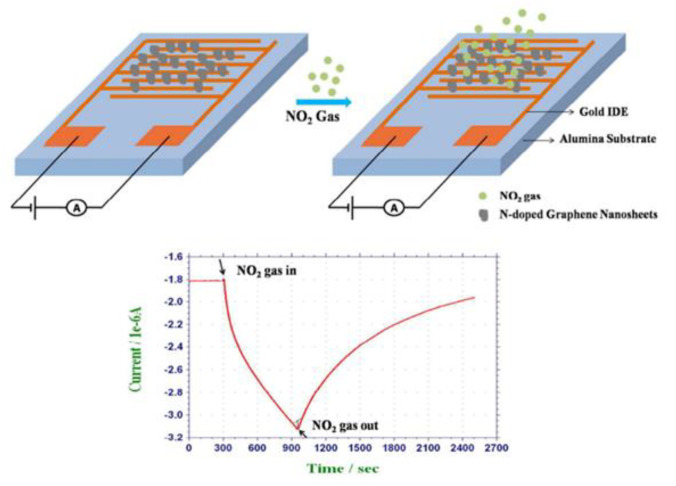
NGS/IDE for detection of NO_2_ gas at room temperature. Reprinted with permission from [74].

The development of sensors employing chemiresistors and field-effect transistors has received a lot of attention since nitrogen dioxide is one of the most prevalent harmful gases. Compounds such as organic materials, carbon nanomaterials, conducting polymers, and semiconducting metal oxides [73] were explored for detecting the existence of NO_2_ gas in the atmosphere. An inert iron (II) phthalocyanine (FePc) thin film-based chemiresistor sensor using FePc and an organometallic molecular crystal was developed for the sensing of nitrogen dioxide [73]. FePc works as an electron donor whenever nitrogen dioxide gas is present and generates a charge carrier complex. This complex decreases the resistivity, and its measurement helps in the estimation of NO_2_ concentrations. Consistently, a concentration in the range of 0.5–2 ppm was recorded. Shaik et al. in 2015 reported chemiresistive sensors utilizing nitrogen-doped graphene nanosheets coated onto the interdigitated electrodes NGS/IDE for the detection of nitrogen dioxide gas at room temperature [74]. The proposed sensor showed good response to low concentrations with a minimal detection threshold of 120 ppb (S/N = 3). Even at high concentrations, the sensor displayed outstanding selectivity for sensing NO_2_ gas in comparison to a variety of interfering gases, including ethanol, CO, H_2_S, NH_3_, dichloromethane (DCM), benzene, and chloroform, which may be related to the strong electron-withdrawing properties of NO_2_ gas. The increased capacity of the NGS/IDE sensor to detect NO_2_ gas could be attributed to the increased accessibility of active sites for the adsorption of gas because of the nitrogen doping in NGS. Figure 14 shows NGS/IDE for the room temperature sensing of NO_2_ gas.

**Figure 15:**
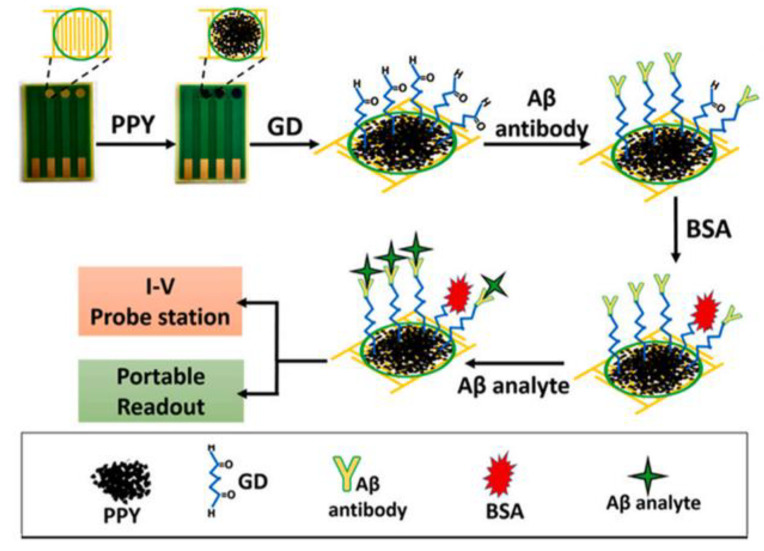
Schematic representation of the proposed Aβ sensing platform. Reprinted with permission from [75].

Chemiresistor sensors are also used for various biosensing applications, such as detection of various nitroaromatic peptides, which can help in the detection of several diseases. Many groups of researchers reported employing polypyrrole nanoparticle-based chemiresistive biosensors to detect Alzheimer’s disease (AD), specific biomarkers Aβ40 and Aβ42 peptides on a unique platform. The suggested platform was able to detect both nitroaromatic peptides over a broad detection range (10^−14^–10^−6^ g/mL), with limited sensing on the order of 10^−15^ g/mL. A schematic representation of the proposed Aβ sensing platform is shown in Figure 15 [75].

## 4. Challenges and Conclusions

NACs are one of the most commonly found pollutants in the environment. In light of the harmful impacts of NACs, many articles in the literature have been published for their regulation and sensing. There is extensive review literature on gas sensing using chemiresistive sensors [73,74]. However, there is none on nitroaromatic compound sensing using chemiresistors. We have discussed chemiresistive sensing of PAHs and persistent organic pollutants that have not been included in any review paper on chemiresistive sensors.

Several materials such as graphite and their composites, carbon nanotubes, and quantum dots are conductive in nature and hence usually used in chemiresistive sensing applications. In the case of nitroaromatic compounds, only nitro explosives such as TNT, DNT, RDX, picric acid (PA), etc. have been explored using various materials, as shown in Table 1. Cross-sensitivity is also a problem that is seen in the sensing application of analytes with nano materials. Cross sensing is a phenomenon of similar responses to different types of analytes [76]. In the case of chemiresistive sensors, low sensitivity and poor selectivity are major challenges [28]. Metal oxides offer greater sensitivity than other materials. Since they work at extremely high temperatures, they have limitations such as baseline drift and limited selectivity. [77]. Apart from metal oxides, carbon-based materials have higher surface area, but they have poor selectivity and low response and reproducibility [78].

In the field of nanomaterials, metal organic frameworks (MOFs) are emerging as materials for sensing applications with good selectivity, sensitivity, and reproducibility. MOFs can be designed according to the need for analyte synthesis. Sensing of formaldehyde with a Co-based MOF (ZIF-67) was first reported using pure MOF-based chemiresistive sensing [79]. MOF-based chemiresistive sensors for the sensing of nitroaromatics are less explored in the literature.

Poor electrical conductivity is a major challenge to the potential use of pure MOFs for applications in chemiresistive sensing. Generally, MOFs are not conductive at ambient temperature. Strong orbital overlap and hard metal ions in MOFs prevent electron transit or circulation [80]. Today, the synthesis of conductive MOFs (C-MOFs) is not a difficult task. Various pre- and post-synthesis approaches are available to produce MOFs as C-MOFs. C-MOFs can effectively transduce the electrical signals from surface processes, making them a new family of substances for chemiresistive sensors. Apart from pure MOF, MOF-based composite materials/MOF derivatives are used in chemiresistive sensor applications.

In the field of nitroaromatic sensing, we have great opportunities to explore chemiresistive sensing applications for different classes of pollutants, such as nitro explosives, nitro polycyclic aromatic hydrocarbons, nitro-based pesticides, and other emerging pollutants.

## Figures and Tables

**Table 1:** Summary of chemiresistive sensing of nitro explosives with different materials.

Type of Sensor	Analyte	Material Used in Sensing	Detection Limit	Analyte Concentration	Response	Reference
Quantum dots	NB	PbS quantum dots	65 ppb–16 ppm	-	-	[31]
Schottky junction-based	DNT	Core-shell ZnO/reduced graphene oxide (rGO)	411 ppb	-	-	[32]
	TNT	Core-shell ZnO/reduced graphene oxide (rGO)	9 ppb	-	-	[32]
	RDX	Core-shell ZnO/reduced graphene oxide (rGO)	4.9 ppt	-	-	[32]
Nanosheets	DNT	TiO_2_ nanosheets	-	180 ppb	65.5%	[33]
	TNT	TiO_2_ nanosheets	-	9 ppb	115.6%	[33]
	RDX	TiO_2_ nanosheets	-	4.9 ppt	40.0%	[33]
	PNT	TiO2 nanosheets	-	647 ppb	830.0%	[33]
	PA	TiO_2_ nanosheets	-	097 ppb	115.0%	[33]
Organic polymer film	TNT	PPy-BCGnBA	0.2 ppb	-	-	[34]
Fibers	TNT	flower-like PANI fibers	0.094 ppb	-	8.1 s	[35]
	PA	flower-like PANI fibers	0.029 ppb	-	-	[35]
Transition-metal doped nanoparticle	TNT	Ni-ZnO	-	9.1 ppb	45.5%	[36]
	DNT	Fe-ZnO	-	411 ppb	38.9%	[36]
	RDX	Ni-ZnO	-	4.9 ppt	45.5%	[36]
	PNT	Ni-ZnO	-	647 ppb	22.9%	[36]
	PA	Fe-ZnO	-	0.97 ppb	36.1%	[36]
SWCNT	TNT	PMA-SWCNT network	-	10 ppt	-	[37]
Carbazole oligomer CNT composite materials	NT (4-nitrotoluene)	Tg-Car/CNT	95 ppb	-	-	[38]

## Data Availability

Not applicable.
